# Nitride Tuning of Magnetic Frustration in the Double Perovskite Ba_2_MnWO_6_

**DOI:** 10.1021/acs.chemmater.4c02114

**Published:** 2024-10-09

**Authors:** Judith Oró-Solé, Carlos Frontera, Jhonatan R. Guarín, Jaume Gàzquez, Bernat Mundet, Clemens Ritter, Josep Fontcuberta, Amparo Fuertes

**Affiliations:** † Institut de Ciència de Materials de Barcelona (ICMAB-CSIC), Campus UAB, 08193 Bellaterra, Spain; ‡ Institut Laue-Langevin, 71 Av. de Martyrs, BP 156, F-38042 Grenoble Cedex 9, France; § Institut Català de Nanociència i Nanotecnologia (ICN2), Campus UAB, 08193 Bellaterra, Barcelona, Spain

## Abstract

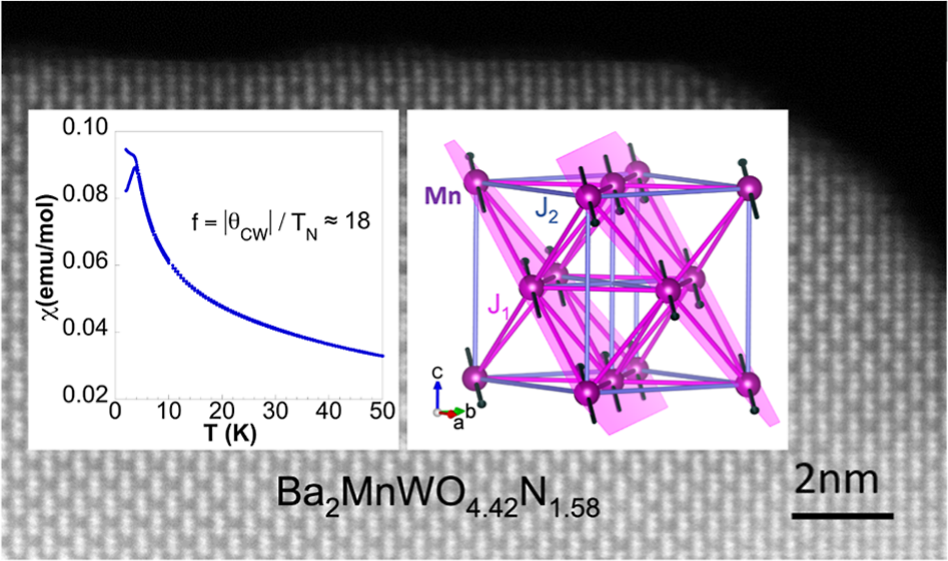

The new double perovskite oxynitride Ba_2_MnWO_4.42_N_1.58_ has been obtained by topochemical ammonolysis at 700 °C of B-site ordered Ba_2_MnWO_6_ using a high NH_3_ flow rate of 600 cm^3^/min. The relatively low synthesis temperature hinders the cation mobility, allowing the order of Mn and W cations of the precursor oxide to be unperturbed in the oxynitride. Synchrotron X-ray diffraction, electron diffraction, and neutron diffraction indicate that Ba_2_MnWO_4.42_N_1.58_ crystallizes in the *Fm*3̅*m* space group with a larger parameter of 8.2434(5) Å compared to Ba_2_MnWO_6_ [8.20337(1) Å]. Magnetic susceptibility measurements show that Ba_2_MnWO_4.42_N_1.58_ orders antiferromagnetically below *T*_N_ = 3.8 K, and the observed Curie–Weiss temperature θ_CW_ = −70(3) K indicates a frustrated Mn lattice with frustration parameter *f* = |θ_CW_|/*T*_N_ ≈ 18, which is significantly larger than for the oxide (*f* ≈ 7.2). The substitution of oxide by the nitride anion induces the oxidation of Mn^2+^ to Mn^3+/4+^ and a subsequent decrease of the paramagnetic effective moment from 6.28 to 5.16 μ_B_/f.u. The observation in the oxynitride of a frustration parameter which is larger than twice that of the oxide precursor is rationalized as caused by the relative enhancement of the nearest neighbors’ magnetic interactions (*J*_1_) Mn–(O/N)–(O/N)–Mn at 90°, compared to next-nearest neighbors’ interactions at 180° (*J*_2_) Mn–(O/N)–W–(O/N)–Mn, due to the smaller electronegativity of nitrogen compared to oxygen that increases the covalency of bonding. These results expand the chemical and structural diversity of complex transition metal oxynitrides and provide a new route to tailor spin frustration in transition metal double perovskites.

## Introduction

**Figure 1:**
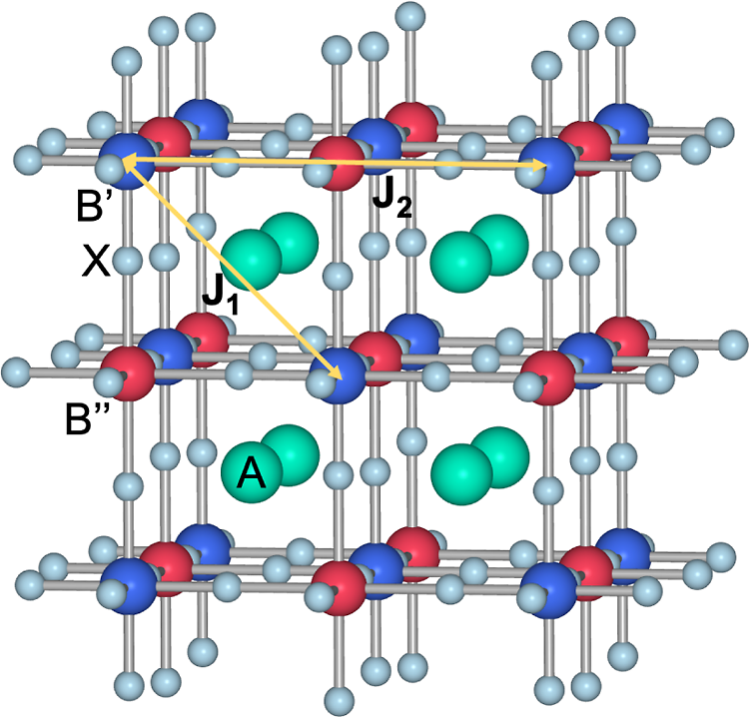
Crystal structure of B site rock salt ordered A_2_B′B″X_6_ transition metal double perovskites with magnetic B′ and diamagnetic B″ cations, showing *J*_1_ and *J*_2_ magnetic interactions between nearest (nn) and next nearest (nnn) B′ cations, respectively.

Double perovskites A_2_B′B″O_6_ with two different transition metals at the B site have been largely investigated because of their wide diversity of electronic, photocatalytic, and ion conducting properties.^1^ The crystallographic order of B′ and B″ cations is a determining factor for the electronic interactions between the transition metals and has a strong influence on the physical properties, most notably on the resulting magnetic behavior. Of particular interest is the case of rock salt ordering of the two cations where B′ is a magnetic ion and B″ is diamagnetic with electron configuration either d^0^ or d^10^. This results in a face centered cubic (fcc) magnetic sublattice for B′, where coexisting *J*_1_ interactions between nearest neighbor (nn) ions and *J*_2_ interactions between next-nearest neighbors (nnn) at 90 and 180°, respectively, may lead to magnetic frustration, compromising the establishment of magnetic order at low temperature (Figure 1).

In the quest of quantum phases of matter, magnetic frustration appears to be instrumental toward spin liquids. In double perovskites such as Sr_2_CuB″O_6_^2^ or Ba_2_MnB″O_6_ (B″ = W, Te),^3,4^ the magnetic frustration is primarily determined by the orbital filling of B″, with 5d^0^ (for W^6+^) or 5d^10^ (for Te^6+^) configurations, by controlling the *F* ≈ *J*_1_/*J*_2_ interaction strength ratio. Cu^2+^ is a Jahn–Teller ion prone to distort the Sr_2_Cu(W, Te)O_6_ structure from the cubic symmetry, hence it could had been guessed that the resulting distortion has a more prominent role on *J*_1_/*J*_2_ than the d^0^ or d^10^ filling. However, the observation of a similar effect in Ba_2_Mn(W, Te)O_6_, where the Mn^2+^ ions have *S* = 5/2 and *L* = 0 configuration, excludes any Jahn–Teller and spin–orbit coupling effects on the *J*_1_/*J*_2_ ratio, strongly supporting its electronic control by orbital filling. In the same vein, it has been recently argued that in the A_2_MnB″O_6_ compounds, increasing tilting of the B′O_6_ and/or B″O_6_ octahedra as a consequence of a reduced tolerance factor results in a decrease of the frustration parameter *J*_1_/*J*_2_.^5^ This view has been challenged by the observation that the oxynitride La_2_Mn^2+^Ta^5+^O_5_N, while displaying a rather small tolerance factor, shows a relatively large frustration parameter (*f* = 6).^5^ This apparent discrepancy was initially attributed to partial ordering of nitrides in the equatorial sites of the Mn octahedra. However, this explanation neglects any electronic role of N in the magnetic interactions, and it is clear that deeper studies are required. Indeed, recent results indicate the extreme sensitivity of magnetic interactions toward the anion composition in oxide and nitride spinels.^6^

Perovskite oxynitrides ABO_3–*x*_N_*x*_ (A = rare earth, B = transition metal) have been reported to show relevant dielectric,^7^ magnetic,^8^ polar,^9^ and photocatalytic^10^ properties that may lead to novel applications. The large covalency of the metal–nitrogen bond, together with the higher formal electrical charge of N^3–^ compared to O^2–^, tune their electronic properties and allow the stabilization of new compounds with the transition metals in higher oxidation states with respect to oxidic analogues, changing the physical properties. Double perovskite oxynitrides A_2_B′B″O_6–*x*_N_*x*_ have been reported for few compounds with Sr or La at the A sites and the combinations B′/B″ = Fe/W,^11^ Fe/Mo,^12,13^ Mg/Ta,^14^ and Mn/Ta,^5^ all of them showing rock salt order in the B sites. Cation ordered oxynitride compounds can be obtained by topochemical nitridation of the respective ordered oxides under gaseous NH_3_ at moderate temperatures^11,12^ or from mixtures of oxides and oxynitrides at high pressure,^5^ whereas the ammonolysis reactions of mixtures of oxides leads to high B′/B″ disorder. Sr_2_FeMoO_5_N with high cation order is ferromagnetic with *T*_c_ ≈ 100 K and shows magnetoresistance below this temperature,^12^ whereas both Sr_2_FeWO_5_N^11^ and La_2_TaMnO_5_N^5^ develop antiferromagnetic interactions between the Fe^3+^ or Mn^2+^ cations below 13 and 4.5 K, respectively.

The rock salt ordered double perovskite Ba_2_MnWO_6_ shows an antiferromagnetic transition with *T*_N_ ≈ 8.8 K and Curie–Weiss temperature θ_CW_ = −63.1 K from coupling of Mn^2+^ spins with *S* = 5/2 in high spin configuration. The ratio between *T*_N_ and θ_CW_ is a measure of the magnetic frustration that for this compound is *f* = |θ_CW_|/*T*_N_ = 8.^3,15^ Aiming at exploring in detail the role of the difference in electronegativities between N and O (χ = 3 and 3.4, respectively^16^) on the magnetic properties in this perovskite -more precisely on the magnetic frustration and ultimately on the magnetic order- we report here the synthesis and study of Ba_2_MnWO_4.42_N_1.58_. The new double perovskite oxynitride shows a high cation order and is prepared by the topochemical reaction of Ba_2_MnWO_6_ with NH_3_ at moderate temperatures. The introduction of nitrogen is accompanied by the oxidation of Mn^2+^ to formally Mn^3+^/Mn^4+^ according to the analyzed anion stoichiometries, and the magnetic interactions remain largely antiferromagnetic (θ_CW_ = −78 K) as in the parent compound Ba_2_MnWO_6_. However, the onset of magnetic order, identified by a peak in the magnetic susceptibility at *T*_N_, is shifted toward lower temperature indicating a magnetic frustration *f* = |θ_CW_|/*T*_N_ ≈ 18, that is larger than twice that of the oxide compound. Careful analysis of the magnetic interactions *J*_1_ and *J*_2_ and the corresponding supersuperexchange pathways allow us to conclude that the introduction of the less electronegative nitrogen atoms in the lattice has distinct effects on *J*_1_ and *J*_2_, the former being reinforced and leading to a large frustration in the oxynitride, as observed. This finding offers a new tool to tune the magnetic interactions in fcc magnetic lattices.

## Experimental Methods

### Synthesis and Chemical Characterization

The oxynitride samples with composition Ba_2_MnWO_6–*x*_N_*x*_ (*x* = 1.36, 1.58) were prepared by topochemical nitridation of Ba_2_MnWO_6_. The double perovskite oxide with high cation order was prepared by solid state reaction between BaCO_3_(Aldrich 99.999%), WO_3_(Aldrich 99.995%), and MnO(Aldrich 99%) under N_2_(Air Liquide, 99.9999%). The three reactants were mixed in stoichiometric ratio, pelletized, and heated at temperatures between 950 and 1250 °C during several treatments with intermediate regrinding for a total time of 174 h, until the sample was free of secondary phases according to X-ray powder diffraction. The sequence of temperatures and treatment times were as follows: two treatments of 15 h at 950 °C, two treatments of 16 h at 1100 °C, two treatments of 16 h and two treatments of 20 h at 1200 °C, and two treatments of 20 h at 1250 °C. Portions of 200 mg of Ba_2_MnWO_6_ in powder form were placed in an alumina boat and treated under NH_3_ flow rate of 600 cm^3^/min at 700 °C using cycles of 12 h with intermediate regrinding. The extent of the dissociation of NH_3_ into H_2_ and N_2_ decreases with increasing the gas flow hence the high rate of 600 cm^3^/min was selected in order to prevent the reduction of the transition metals that could result into the decomposition of the double perovskite oxynitride. The number of cycles were 11 for the sample with *x* = 1.36 and 15 for the sample with *x* = 1.58, and the total reaction times were 132 and 180 h, respectively. A mixture of BaMnO_3_ and BaWO_4_ in proportion 1:1 prepared by a citrate sol–gel method was also used as a precursor sample for the ammonolysis reaction. For the preparation of 1 g of Ba_2_WMnO_6_, stoichiometric amounts of Ba(NO_3_)_2_ (Aldrich 99%), and Mn(CH_3_COO)_2_. 4 H_2_O (Aldrich 99%), and (NH_4_)_6_H_2_W_12_O_40_. 3H_2_O (Aldrich 99.99%) were dissolved in c.a. 30 ml of H_2_O, and an excess of 7 g of C_6_H_8_O_7_ (Aldrich 99.5%) was subsequently added. The solution was evaporated at 80 °C with continuous stirring during c.a. 10 hours, and the resulting sample was heated slowly from 300 to 800 °C under N_2_, with treatments of 0.5 h at a constant temperature every 100 °C. The ammonolysis reaction of portions of 200 mg of this sample was performed at 650 °C under NH_3_ flow rate of 600 cm^3^/min with several treatments, during a total time of 52 h. Handling of the oxynitride samples for further characterization was performed in a glovebox under a recirculating Ar atmosphere.

N contents were determined by combustion analysis in a Thermo Fisher Scientific instrument, heating the samples in oxygen up to 1060 °C and using MgO, WO_3_, and Sn as additives and atropine as a reference standard.

### Structural Characterization

X-ray powder diffraction data were acquired on a Panalytical X’Pert Pro MPD diffractometer with Cu Kα radiation (λ = 1.5418 Å), and for capillary samples, on a Bruker D8 Advance A25 diffractometer in a Debye–Scherrer configuration with Mo Kα_1_ radiation (λ = 0.7093 Å). High resolution synchrotron X-ray powder diffraction (SXRPD) data were collected at room temperature on capillary samples in the angular range 2.0° ≤ 2θ ≤ 60° at the MSPD beamline^17^ of the ALBA Synchrotron (Cerdanyola del Vallès, Spain), using 30 keV energy that resulted in exact wavelength of 0.41385 Å determined by refining SRM640d NIST Si standard. Neutron powder diffraction data of the oxynitride with composition Ba_2_MnWO_4.42_N_1.58_ were collected in the D20 diffractometer at the Institut Laue Langevin (ILL), on a 150 mg sample that was placed in a vanadium can sealed with indium inside the glovebox. For the magnetic structure determination data, a high flux and low-resolution configuration was used, with a pyrolytic graphite monochromator and a low take off angle (42°) producing neutrons with a relatively large wavelength (λ = 2.44 Å). For the crystal structure determination, a high resolution mode was used with a Ge monochromator and high take off angle (118°) that produced neutrons with a wavelength of 1.36 Å. Rietveld analysis was performed with Fullprof.^18^ The data were corrected for absorption, and background refinement was performed by linear interpolation.

Electron diffraction micrographs were obtained in a JEOL1210 transmission electron microscope operating at 120 kV. The reconstruction of the reciprocal lattice of the samples was performed by tilting the crystals using a double tilt ±60°/±30° specimen holder. The samples were prepared by depositing the powder on a copper grid coated with a holey carbon film. The microstructure of the samples was investigated by scanning transmission electron microscopy (STEM) at the Joint Electron Microscopy Center at ALBA (Cerdanyola del Vallès, Spain) on a double-corrected ThermoFisher Spectra 300 (S)TEM microscope operated at 300 kV, equipped with a monochromator and a continuum spectrometer with a direct electron detection camera (K3). High-angle annular dark field images were acquired using a semiconvergence angle of 19.5 mrad. Electron energy loss spectroscopy (EELS) spectra were collected using a collection angle of around 21.4 mrad, a probe current of around 90 pA, and a dwell time of 50 ms. A principal component analysis filter was used after acquisition to minimize the random noise of the EELS spectrum images. EDX spectra were acquired using a four quadrant Super-X windowless silicon drift detector system and beam currents of ≈100–250 pA. The crystal composition was quantified using the Thermo Fisher Scientific Velox software, applying the Cliff–Lorimer approach with Brown–Powell ionization cross-section models.

### Magnetic Measurements

Magnetic measurements were performed at fields between 50 Oe and 5 kOe and temperatures from 2 to 300 K, using a Quantum Design SQUID magnetometer. Magnetization-field loops were measured between −70 and +70 kOe between 2 and 50 K.

## Results and Discussion

### Topochemical Nitridation of Ba_2_MnWO_6_ and Structural Study of Ba_2_MnWO_6–*x*_N_*x*_ Double Perovskites

**Figure 2:**
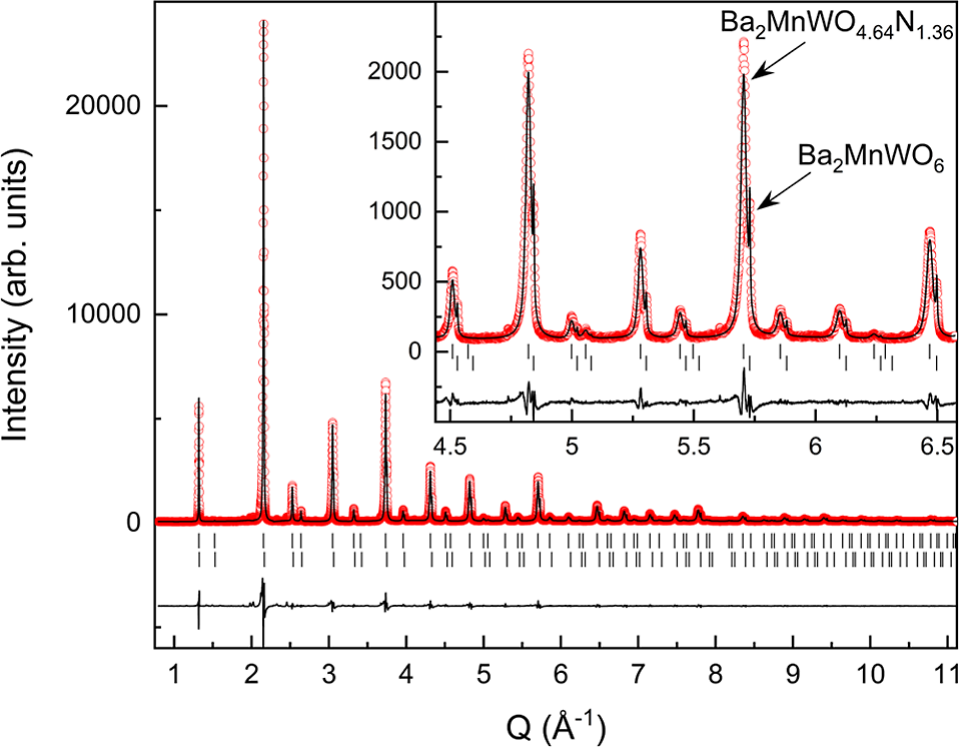
Rietveld fit to SXRPD pattern at room temperature of Ba_2_MnWO_4.64_N_1.36_, performed in space group *Fm*3̅*m* with *a* = 8.24144(4) Å. Upper and lower reflection markers are, respectively, for Ba_2_MnWO_4.64_N_1.36_ and Ba_2_MnWO_6_. The inset shows an enlarged 2θ region with peaks of the two phases indicated by arrows.

**Figure 3:**
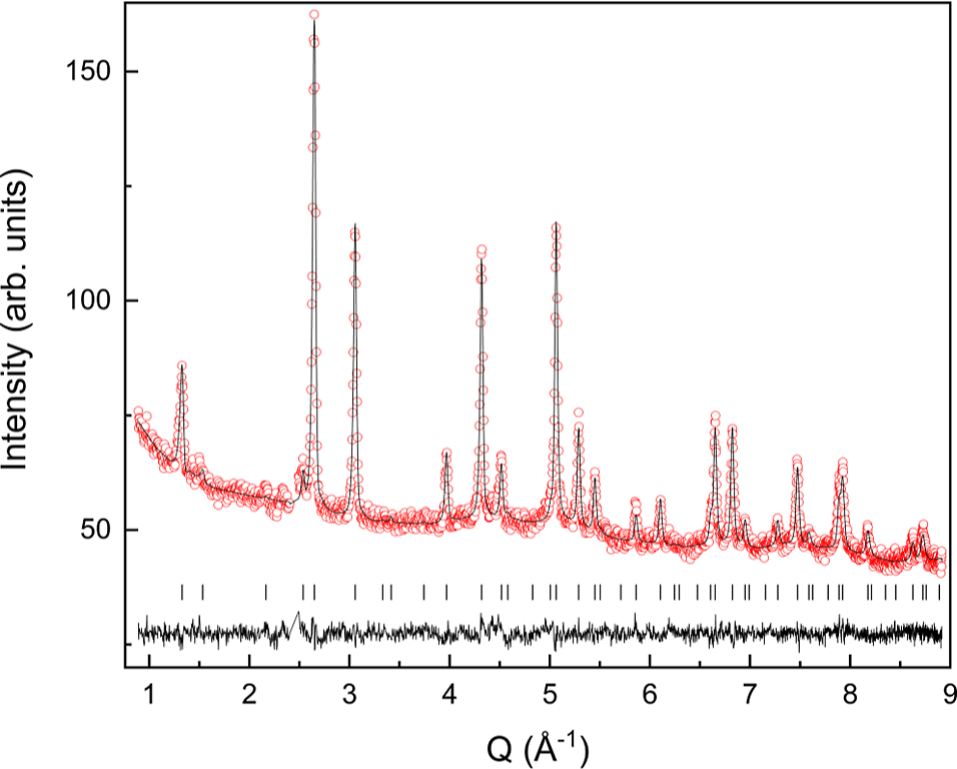
Rietveld fit to neutron powder diffraction pattern at room temperature of Ba_2_MnWO_4.42_N_1.58_, performed in space group *Fm*3̅*m* with *a* = 8.2434(5) Å.

The synthesis under NH_3_ of Ba_2_MnWO_6–*x*_N_*x*_ was performed starting with a precursor oxide sample that was prepared by using two different methods. Highly crystalline Ba_2_MnWO_6_ was obtained by solid state reaction between BaCO_3_, MnO, and WO_3_ in N_2_ using several treatments at temperatures up to 1250 °C. An alternative citrate synthesis method was used to prepare a more reactive oxide precursor consisting of a 1:1 mixture of the hexagonal perovskite BaMnO_3_ and the scheelite BaWO_4_ that was obtained under N_2_ at a maximum temperature of 800 °C (See Figure S1). Rietveld refinement of the X-ray diffraction data of Ba_2_MnWO_6_ was performed in the *Fm*3̅*m* model of the double perovskite structure with Ba at the 8*c* sites and variable occupancies of W and Mn in the positions 4*a* and 4*b*, respectively, and converged to antisite disorder (the fraction of Mn in 4*a* or W in 4*b* site) of 0% and *a* = 8.20337(1) Å (Figure S2 and Table S1). The ammonolysis of both oxidic precursors was performed at temperatures between 650 and 750 °C under an NH_3_ flow rate of 600 cm^3^/min. The treatments above 700 °C led the decomposition of the double perovskite, whereas below this temperature, the kinetics of the nitridation was very slow in the case of the oxide prepared by solid state reaction. The reaction with NH_3_ of the two precursors produced the formation of the oxynitride Ba_2_MnWO_6–*x*_N_*x*_ with analyzed nitrogen contents up to 2 atoms per formula unit. The oxygen stoichiometries were calculated by difference, assuming that the total anion content was six atoms per formula. The nitridation of the mixture of oxides prepared by the citrate method was performed at 650 °C with several treatments of total time of 52 h and proceeded through the formation of the intermediate compounds Ba_3_W_2_O_6_N_2_ and Ba_3_WN_4_. The samples changed with nitridation from yellowish green for Ba_2_MnWO_6_ to greenish brown for the oxynitrides. The refinement of the synchrotron X-ray diffraction pattern of the resulting oxynitride with composition Ba_2_MnWO_4_N_2_ (Figure S3) returned the parameter *a* = 8.23911(5) Å and a high antisite disorder of 19.2%. In contrast, the ammonolysis of Ba_2_MnWO_6_ at 700 °C produced topochemically the direct formation of a B-site ordered double perovskite oxynitride, coexisting with the oxide, in a proportion that increased progressively with the treatment time. The SXRPD pattern of a sample obtained after 132 h under NH_3_ with nitrogen content of 1.36(3) atoms per formula indicated the presence of the oxynitride as major phase, and a small fraction (5% w/w) of Ba_2_MnWO_6_. The reconstruction of the reciprocal lattice by electron diffraction was performed for several crystals in this sample that showed similar crystallographic planes to those of the pristine Ba_2_MnWO_6_, with reflection conditions compatible with the space group *Fm*3̅*m* (See figure S4). Rietveld refinement of the SXRPD data (Figure 2 and Table S2) determined *a* = 8.24144(4) Å for the oxynitride phase with stoichiometry Ba_2_MnWO_4.64_N_1.36_, which showed broader peaks that the pristine Ba_2_MnWO_6_ and larger cell parameter [*a* = 8.20511(3) Å for Ba_2_MnWO_6_ in this sample]. The refined antisite disorders were 2.59% for oxynitride and 0% for oxide, indicating some cation mobility during nitridation. Four subsequent treatments of Ba_2_MnWO_4.64_N_1.36_ under NH_3_ in the same conditions increased the nitrogen content to 1.58(3) atoms per formula, and decreased the proportion of Ba_2_MnWO_6_ to 1.38% as determined from the refinement to SXRPD data. The composition of several single crystals of this sample was investigated by EDX with atomic resolution using STEM. The observed atomic ratios Ba:Mn:W:O:N = 1.93(5):0.86(5):0.86(9):5.1(4):1.3(3) were in rough agreement with the stoichiometry Ba_2_MnWO_4.42_N_1.58_ indicated by chemical analysis, considering the standard deviations. Rietveld fit to neutron powder diffraction data of Ba_2_MnWO_4.42_N_1.58_ (Figure 3 and Table 1) performed in the space group *Fm*3̅*m* with N/O in the 24*d* site led to a = 8.2434(5) Å and antisite disorder of 5%. Rietveld refinements from both synchrotron X-ray diffraction and neutron diffraction data were also performed in the tilted *I*4*/m* structure of the double perovskite, where there are two crystallographically independent anion positions that could be occupied by N and O in different proportions. However, the agreement factors for this space group were significantly larger than those obtained for the *Fm*3̅*m* model, which shows one single anion site occupied by N and O in a disordered manner. The observed W–N/O bond distance in Ba_2_MnWO_4.42_N_1.58_ was 1.947(4) Å, slightly larger than in Ba_2_MnWO_6_, 1.923(3) Å, consistent with the larger ionic radius of N^3–^ compared to O^2–^ [for CN = IV *r*(N^3–^) = 1.46 Å and *r*(O^2–^) = 1.38 Å].^19^ This effect is also observed when comparing the Ba–O,N [2.917(3) Å in Ba_2_MnWO_4.42_N_1.58_] and Ba–O [2.903(2) Å in Ba_2_MnWO_6_] bond distances. In contrast, the observed Mn–O, N bond distance, 2.175(4) Å, is similar to the Mn–O distance in Ba_2_MnWO_6_ [2.179(3) Å], indicating that for this bond, the increase caused by the incorporation of nitride is compensated by the reduction of the ionic radius of Mn as a consequence of the oxidation of this cation,^19^ according to the charge balanced formal stoichiometry Ba_2_Mn^3+^_0.42_Mn^4+^_0.58_W^6+^O_4.42_N_1.58_.

**Table 1:** Summary of the *Fm3̅m* Model Refined Against Room Temperature Neutron Powder Diffraction Data for Ba_2_MnWO_4.42_N_1.58_ (λ = 1.36 Å)^a^

atom	site	*x*	*y*	*z*	*B* (Å^2^)	occupancy
Ba	8*c*	0.25	0.25	0.25	1.2(2)	1
Mn1/W1	4*b*	0.5	0.5	0.5	0.19(10)	0.949(4)/0.051
W2/Mn2	4*a*	0	0	0	0.19	0.949/0.051
O/N	24*d*	0.2362(3)	0	0	1.23(6)	0.736/0.264

bond	*d* (Å)	bond	*d* (Å)	bond	*d* (Å)
Ba–O	2.917(3) × 12	Mn1/W1–O/N	2.175(4) × 6	W2/Mn2–O/N	1.947(4) × 6

a The O/N occupancies were constrained to the anion composition obtained by chemical analysis. Refined cell parameter and agreement factors: *a* = 8.2434(5) Å. *R*_Bragg_ = 4.48%, *R*_wp_ = 2.21%.

**Figure 4:**
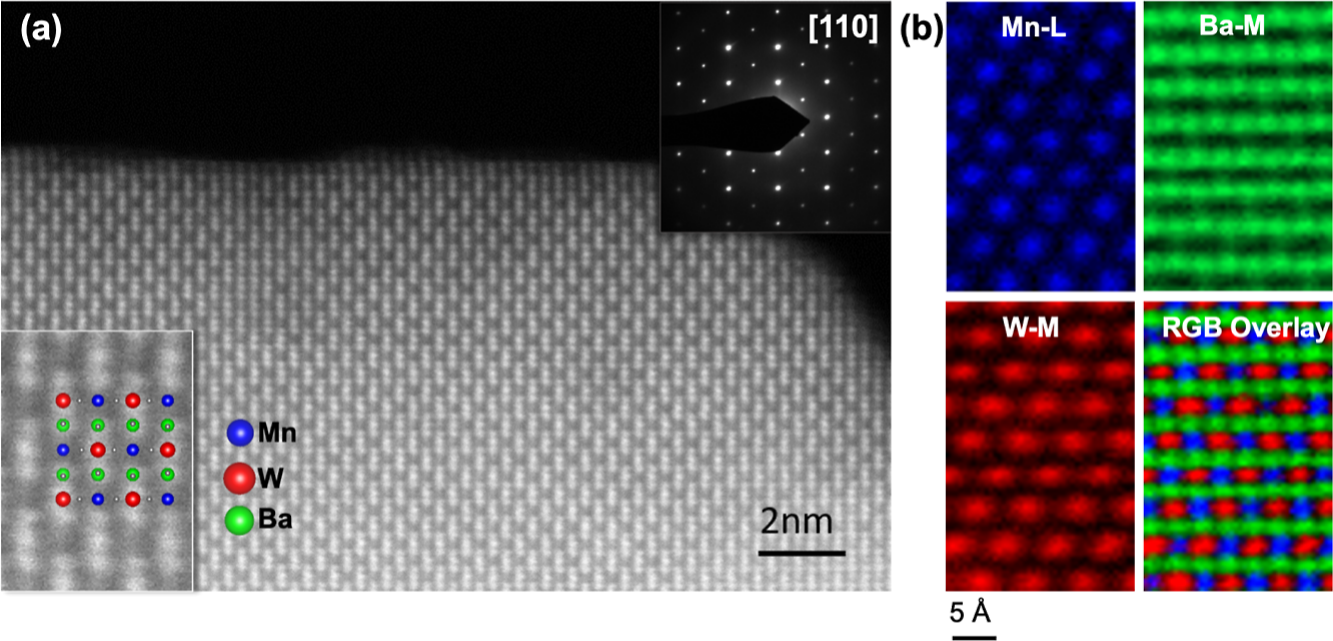
(a) Z-contrast image of the Ba_2_MnWO_4.42_N_1.58_ structure along the [110] axis. Insets show a higher magnification image with the structure superimposed (bottom left) and an electron diffraction pattern (top right corner). (b) EELS elemental maps of the Mn L-edge, Ba M-edge, and W M-edge signals and the corresponding RGB map, respectively. The latter is the overlay of each elemental map, with Mn in blue, Ba in green, and W, in red.

EELS in conjunction with the Z-contrast imaging technique in the scanning transmission electron microscope were used to gather detailed information on the composition, chemistry, and structure of the oxynitride with atomic resolution. Figure 4a shows the Z-contrast image of the Ba_2_MnWO_4.42_N_1.58_ structure along the [110] zone axis. The analyzed crystal is of great quality, without lattice defects or atomic distortions either within the bulk or close to the crystal surface. Notice that the Mn atomic column is hardly seen in the Z-contrast image as both Ba and W have much higher atomic numbers and, therefore, much higher column intensities than the Mn ones. However, one can easily retrieve the perfect ordered structure and composition with EELS imaging, as shown in Figure 4b, in which the Mn, Ba, and W elemental maps are shown in blue, green, and red, respectively, along with their overlaid image. Now, the presence of Mn atoms is obvious, occupying their expected lattice sites. The checkerboard distribution of Mn and W cations in the B site of the perovskite structure shown by the elemental maps unveils the expected highly ordered structure of the Ba_2_MnWO_4.42_N_1.58_ crystal.

### Magnetic Properties

The magnetic properties of the pristine Ba_2_MnWO_6_ (BMWO) and the oxynitride Ba_2_MnWO_4.42_N_1.58_ (BMWON) were analyzed by using dc Squid magnetometry, in the temperature range 2–300 K under various magnetic fields (50 Oe–5 kOe) and when appropriate zero-field-cooled and field-cooled (ZFC-FC) processes where employed.

**Figure 5:**
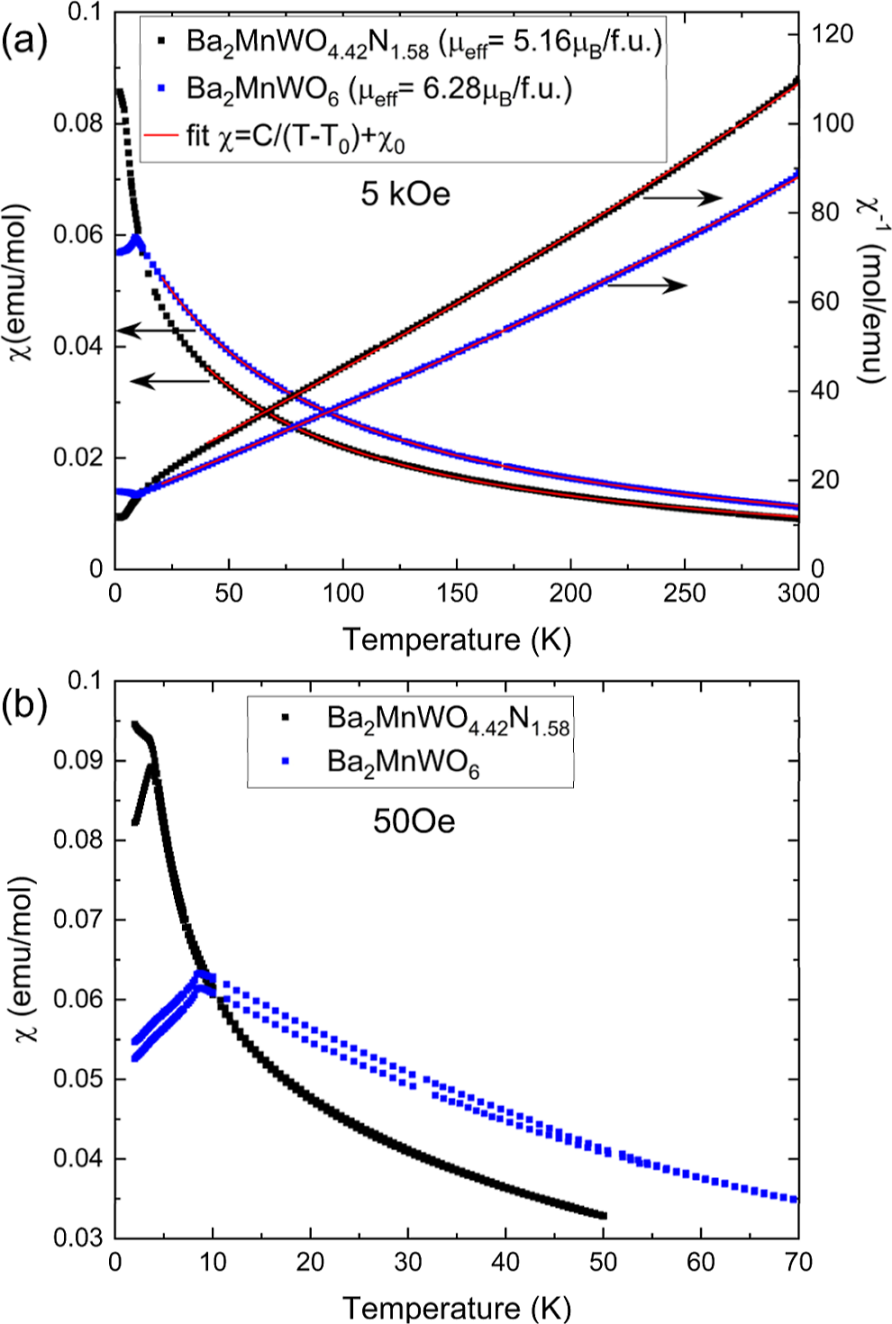
(a) Susceptibility (left axis) and inverse of the susceptibility (right axis) of BMWO (blue squares) and BMWON (black squares) measured under an applied magnetic field of 5 kOe. Red lines plot the fit to the expression: . (b) ZFC-FC susceptibility measured for both samples under 50 Oe of magnetic field.

We first focus on the temperature-dependent susceptibility χ(*T*) and its reciprocal χ^–1^(*T*) recorded at a relatively large magnetic field (5 kOe). Data for BMWO and BMWON are depicted in Figure 5a. It can be appreciated that in the high temperature region, χ(*T*) displays roughly a Curie–Weiss behavior , where θ_CW_ is the extrapolated Curie–Weiss temperature, C is the Curie constant, which is directly related to the paramagnetic magnetic moment (μ_eff_), and χ_0_ accounts for the diamagnetic contributions. The solid red lines through the data correspond to the fits of χ(*T*) and χ^–1^(*T*) in the temperature range of 30–300 K. Data fitting allows us to extract μ_eff_ (BMWO) = 6.28 μ_B_ and μ_eff_ (BMWON) = 5.16 μ_B_. A detailed analysis and justification of the selected temperature range for data fitting can be found in Section S1 of Supporting Information. The effective magnetic moment of BMWO is similar, although slightly larger, to that expected for 3d^5^ (*S* = 5/2) paramagnetic Mn^2+^ ions (μ_eff_ = 5.95 μ_B_) in the formal composition Ba_2_Mn^2+^W^6+^O_6_. For BMWON, any oxidation of Mn^2+^ should reduce the electron counting in the 3d orbitals below 3d^5^, and thus the magnetic moment is expected to be smaller, in agreement with observed 5.16 μ_B_. However, this effective magnetic moment is definitely larger than that expected within the simplest ionic picture in stoichiometric Ba_2_ Mn^3+^_0.42_Mn^4+^_0.58_W^6+^O_4.42_^2–^N_1.58_^3–^ [calculated μ_eff_ (BMWON) = 4.33 μ_B_]. On the other hand, the extrapolated negative Curie–Weiss temperatures θ_CW_ (BMWO) = −75 K and θ_CW_ (BMWON) = −70 K indicate the prevalence of antiferromagnetic interactions between the magnetic Mn^m+^ (*m* = 2, 3, or 4) ions, somewhat reduced in the oxynitride. Interestingly, the two compounds display a low-temperature magnetic feature, namely, a susceptibility kink at χ(*T*_N_). This can be better appreciated in the susceptibility data recorded at lower magnetic field, as shown in Figure 5b, where we identify: *T*_N_ (BMWO) ≈ 8.8 K. Remarkably, and in spite that θ_CW_ in both compounds are rather similar, in the oxynitride, *T*_N_ (BMWON) ≈ 3.8 K occurs at much lower temperature. At *T* < *T*_N_, the susceptibly somewhat decreases indicating the onset of a long-range magnetic order. The formation of a magnetic order at *T*_N_ ≪ θ_CW_, suggests the existence of magnetic frustration, typically quantified via *f* = |θ_CW_|/*T*_N_ that in this case are *f* (BMWON) = 18.4 and *f* (BMWO) = 8.5. Despite the large frustration in the oxynitride, neutron diffraction data have evidenced the existence of a long-range magnetic order at 1.6 K (details are given below). While the existence of magnetic frustration in Mn double perovskites is well documented (see for instance refs (4 and 5) for recent reviews), inspection of available data shows that in the reported (Ba, Sr, La)_2_B′B″X_6_ (X = O, N) compounds B′ = magnetic ion (Mn, Co) and B″ = diamagnetic ion (W, Te, Ta), the frustration parameter is bounded to *f* < 8–9. The observation of *f* ≈ 18.4 in our BMWON sample demonstrates how nitridation reinforces dramatically the frustration, as we will discuss later.

In Figure 5b, well visible ZFC-FC divergences can be appreciated when susceptibility is measured at low field (here, 50 Oe) in both compounds, although they are no longer perceptible in data recorded at high magnetic field (Figure 5a). This is consistent with earlier data on BMWO where no hysteresis was perceived at 1 kOe.^3^ In our BMWO sample, divergence is recognizable below ≈50 K and across *T*_N_ to the lowest temperature, while in BMWON, the divergence is only apparent at *T* < *T*_N_. Divergent ZFC-FC curves imply the presence of ferromagnetic hysteresis and thus that of uncompensated or canted antiferromagnetic magnetic order. In fact, the magnetization loops of the BMWON samples (*x* = 1.36 and 1.58), at the lowest temperature (*T* < 10 K), display a conspicuous curvature, less perceptible in the oxide BMWO, indicating a ferromagnetic behavior (See Figure S5), that cannot be attributed to a paramagnetic response (See Figure S6).

In some manganese oxides, such as the much studied MnO which displays a modest frustration (*f* ≈ 5), antiferromagnetic long-range magnetic order sets in at *T*_N_ = 118 K, but magnetic correlations persist up to much higher temperatures (≈1100 K).^20^ These short-range magnetic correlations in the paramagnetic phase appear to be ubiquitous in frustrated systems and consistently they have been recognized in double perovskites,^2^ more specifically in Ba_2_MnWO_6_ where they have been recently mapped by inelastic neutron scattering.^3^ Consequently, ZFC-FC divergence in BMWO can be attributed to spin-canted, short-range magnetic correlations. However, it is worth noticing that the Mn double perovskite oxide samples may contain tiny concentrations of ferromagnetic Mn_3_O_4_, a byproduct that can be hardly avoided and remains virtually undetectable by diffraction techniques. The presence of this phase could produce similar fingerprints in χ(*T*),^4,21^ than those we tentatively attribute here to short-range magnetic correlations, persisting at high temperature, in agreement with recent findings in La_2_MnTaO_5_N.^5^

**Figure 6:**
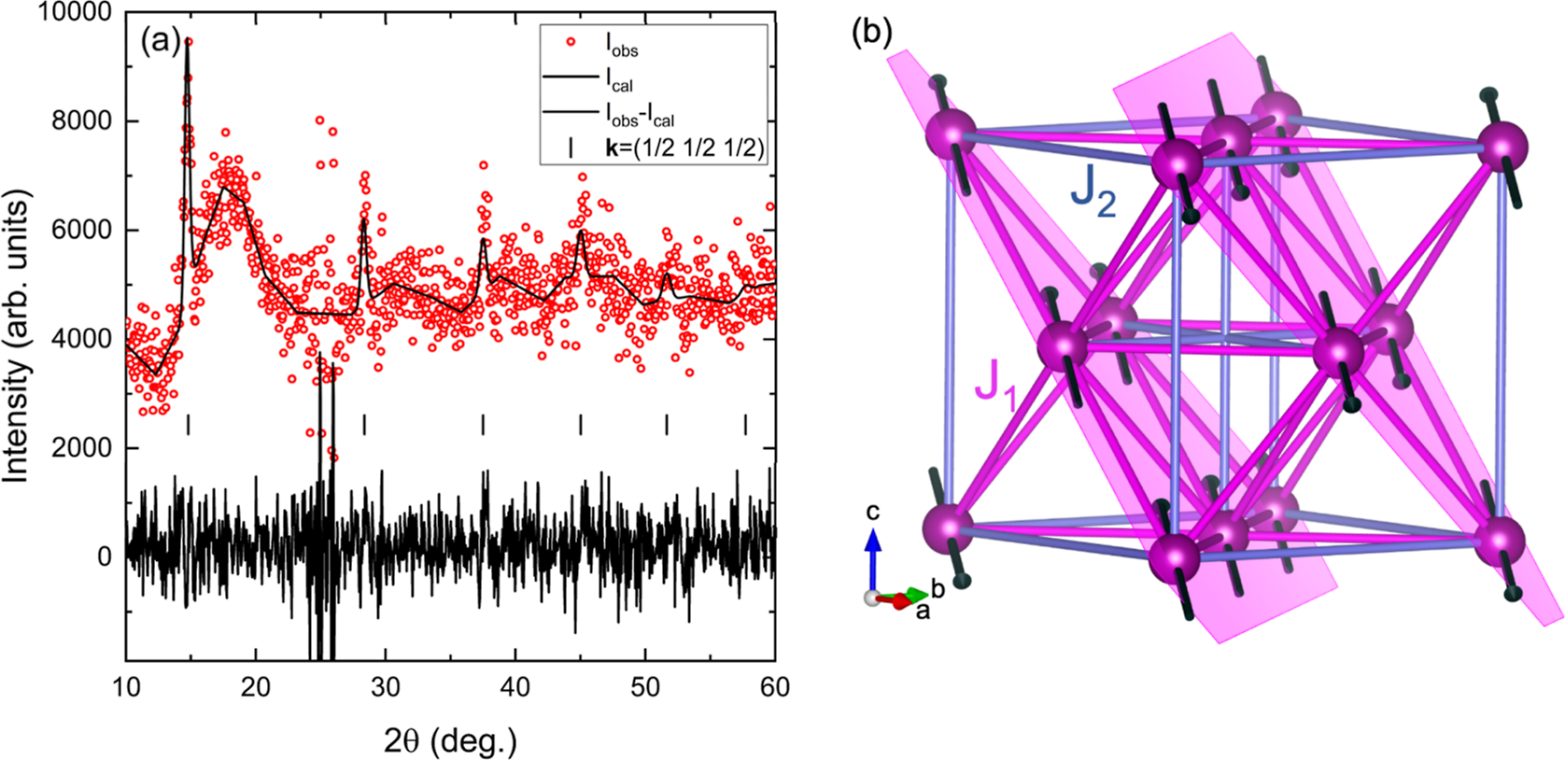
(a) Rietveld refinement of the magnetic diffraction data (λ = 2.4 Å) of Ba_2_MnWO_4.42_N_1.58_ obtained by subtracting the neutron diffraction pattern collected at 10 K from the pattern collected at 1.6 K. Magnetic reflections (marked as |) are generated by the propagation vector **k**= (1/2,1/2,1/2). (b) Refined magnetic structure (only magnetic Mn atoms are depicted) consisting of ferromagnetic [111] planes (purple) coupled antiferromagnetically.

The magnetic structure of Ba_2_MnWO_4.42_N_1.58_ was determined from neutron diffraction intensity data collected at temperatures below (1.6 K) and above (10 K) *T*_N_ at the D20 instrument of ILL, using the high-flux mode (see experimental section). The difference between the two patterns (Figure 6, red symbols) shows a series of diffraction peaks of magnetic origin that can be indexed using the cubic lattice parameter and the *Fm3̅m* space group with the propagation vector **k** = (1/2, 1/2, 1/2), indicating the emergence of a low temperature antiferromagnetic order. Symmetry analysis using the program Basireps for the Mn site 4*b* in space group *Fm3̅m* and **k** = (1/2,1/2,1/2) returned two allowed irreducible representations (IR) with one, respectively, two basis vectors. Only one of these IR was able to refine correctly the magnetic diffraction intensities. The resulting magnetic structure is similar to that of MnO, with (111) ferromagnetic planes coupled antiferromagnetically (Figure 6b). The diffraction peaks are quite narrow, indicating that the spatial range of the magnetic order is high: no significant enlargement of the magnetic diffraction peaks in comparison with the nuclear peaks can be detected, meaning that the size of the magnetic domains is comparable to the crystalline coherence length. However, at 1.6 K the ordered magnetic moment of the Mn ions is much smaller [0.48(1)μ_B_] than the expected magnetic moment of Mn^+3.58^ corresponding to the stoichiometry Ba_2_MnWO_4.42_N_1.58_. As visible in Figure 6, the difference data 1.6–10 K display the main sharp magnetic diffraction peaks as well a broad feature at about 2θ = 18° corresponding to a *d*-spacing of about 8 Å which could be caused by magnetic short-range correlations along the six equivalent directions of the cubic unit cell having *a* ∼ 8 Å. The correlation length, estimated as the inverse of the full width at half-maximum (fwhm) of this peak when data are plotted against 1/*d*, is ∼30 Å. In any case, the low value of the magnetic moment clearly points to a highly frustrated magnetic sublattice.

Data description reported above convey three main messages: (A) μ_eff_ of the pristine compound (BMWO) is larger than that expected from spin-only Mn^2+^ ions, (B) the effective paramagnetic moment of the oxynitride is significantly larger than that expected from the stoichiometry and electron counting, and–of major importance here: (C) the frustration parameter of the oxynitride doubles the frustration parameter reported in related double perovskites. We discus in the following these observations and their implications.

#### Enhanced Paramagnetic Moment in Ba_2_MnWO_6_

We first notice that observation of larger-than-expected μ_eff_ in the double perovskite A_2_Mn^2+^B″O_6_ (B″ = W, Te) has already been reported, although its significance has not been discussed to any extent. Examples are given in Table 2.

**Table 2:** Effective Magnetic Moments of Mn Double Perovskites with Diamagnetic Cations at B″ Sites with d^0^ and d^10^ Electron Configurations, Obtained by Using the Indicated (H-used) Magnetic Field

compound	d^0^/d^10^	μ_eff_ (μ_B_)	H-used (kOe)	reference
Ba_2_MnWO_6_	d^0^	6.28	5	this work
Ba_2_MnWO_6_	d^0^	6.3(3)	1	(3)
Sr_2_MnWO_6_	d^0^	6.2	1	(27)
Ba_2_MnTeO_6_	d^10^	6.30(1)	1	(4)
Sr_2_MnTeO_6_	d^10^	6.05	1	(21)

Mn^2+^ has a 3d^5^ electronic configuration, spin-only (*S* = 5/2, *L* = 0), therefore, we cannot anticipate any angular momentum contribution to μ_eff_. Moreover, any covalency of Mn–O bonds is expected to reduce, rather than to enhance, the magnetic moment of Mn^2+^ ions;^22^ for instance, in the case of MnO, μ_eff_ is reduced by about 3%, to 5.73 μ_B_.^23^ A possible mechanism to enhance the magnetic moment in Ba_2_MnWO_6_ is to invoke the covalency effect of the W–O bonds (a discussion on covalency effects on magnetic properties of related W oxides is included in Section S2 of Supporting Information).^24−26^ Indeed, ab initio, many body quantum chemistry calculations have shown that there is a considerable hybridization W(5d)–O(2p) that results in charge transfer, thus creating unpaired spins at 5d and 2p orbitals.^2^ Under the measuring magnetic field, it can be envisaged that these covalency- related electronic spins should contribute to the measured magnetization leading to an abnormally large μ_eff_. Within this simple picture, the covalency-induced magnetic moment, accounting for the difference: μ_eff_ (BMWO) (=6.28 μ_B_) and μ_eff_ (expected) (=5.95 μ_B_), is of about Δμ ≈ 1.85 μ_B_/f.u (S≃ 1/2), that would roughly correspond to a W–O charge transfer of c.a. 37%. Not surprising, the W–O bond covalency is found to be much larger than the corresponding one for the Mn–O bond.

#### Effective Paramagnetic Moment of the Oxynitride is Significantly Larger than that Expected from the Stoichiometry and Electron Counting

In the first approach, the experimental observation that μ_eff_ (BMWON) = 5.16 μ_B_ is larger than that expected within the simplest ionic picture in stoichiometric Ba_2_ Mn^3+^_0.42_Mn^4+^_0.58_W^6+^O_4.42_N_1.58_ [μ_eff_ (BMWON) = 4.33 μ_B_], could be seen as an indication that the Mn cations are less oxidized than predicted from charge compensation. As W^6+^ cannot be further oxidized and chemical analysis and neutron diffraction guarantee that nitride has been successfully inserted into the structure, it follows that to preserve charge neutrality, the existence of some anion deficiency should be considered. The observed effective magnetic moment of c.a. 5.1 μ_B_/Mn corresponds to about 20% of Mn^2+^ (*S* = 5/2) and 80% of Mn^3+^(*S* = 2), that according to the O/N experimental ratio would be consistent with 21% of anion vacancies [i.e., with the stoichiometry Ba_2_MnW(O_4.42_N_1.58_)_0.79_]. If we consider the W–O covalency effects, as we have already identified in the BMWO compound, then the effective moment coming from Mn^3+/4+^ is estimated to be μ_eff_ = 4.75 μ_B_/Mn, corresponding to about 84% of Mn^3+^ and 16% of Mn^4+^, which implies the presence of a smaller proportion of anionic vacancies: Ba_2_MnW(O_4.42_N_1.58_)_0.88_ (i.e., 12% of anionic vacancies). The introduction of this small amount of vacancies in the refinement of neutron diffraction data leads to an increase of the Bragg R factor from 4.67 to 4.97%, suggesting that the anion sites are fully occupied. Alternatively, one may wonder whether, in an ionic picture, nominal N^3–^–Mn^3+/4+^–O^2–^ and N^3–^–W^6+^–O^2–^ bonds should be expected in BMWON. However, this ionic picture may be challenged by the already mentioned covalency of bonds that will be enhanced in the oxynitride because of the lower electronegativity of nitrogen compared to oxygen. It follows that the electron distribution in N↔Mn and N↔W bonds should be more shifted to the right as compared to the ionic picture, thus increasing the measured effective moment of the observed Mn^m+^ species, which is in agreement with experimental data.

#### Frustration Parameter of the Oxynitride Doubles the Reported Frustration Parameters of Related Double Perovskites

The frustration parameter in BMWON is dramatically enhanced with respect to reported values for related Mn double perovskites, implying that nitriding reinforces the competing interactions within the magnetic Mn cations. In the double perovskites A_2_MnB″X_6_, J_1_ corresponds to nearest-neighbors (nn) Mn–X–B″–X–Mn (90°) supersuperexchange interactions connecting the Mn atoms along the diagonal of the fcc faces (Figures 1 and 6b), whereas *J*_2_ corresponds to next-nearest-neighbors (nnn) interactions Mn–X–B″–X–Mn (180°) along the edges of the A_2_MnB″X_6_ unit cell. For Mn^2+^ the superexchange interactions predicted by Goodenough-Kanamori rules are antiferromagnetic both at 180 and 90°. If *J*_2_ is dominant (|*J*_2_/*J*_1_| > 1), an antiferromagnetic long-range magnetic order in which each magnetic moment is coupled antiferromagnetically to its nnn neighbors can be established at some low temperature. This is the order observed in MnO, having also a fcc structure,^28^ and that reported for Ba_2_MnWO_6_.^3^ However, if *J*_1_ becomes increasingly dominant, the situation is totally different, as not all *J*_1_ antiferromagnetic nn interactions can be fulfilled; then frustration arises and the long-range magnetic order is compromised. Frustration is thus related to the relative values of *J*_1_ and *J*_2_ supersuperexchange interactions (assuming all being antiferromagnetic), that is *F* ≈ *J*_1_/*J*_2_. For Ba_2_MnWO_6_, recent inelastic neutron scattering experiments and magnetization experiments by Mutch et al.^3^ have allowed to determine: *J*_1_ = −0.080 meV and *J*_2_ = −0.076 meV, leading to *F* ≈ 1.06, and *f* = |θ_CW_|/*T*_N_ ≈ 8. It is worth noticing, that similarly to MnO, Much et al. also noticed in Ba_2_MnWO_6_ the persistence of short-range magnetic correlations at *T* > *T*_N_, that may be reminiscent of the similar behavior observed in MnO. The observation of a similar frustration parameter in our BMWO sample, suggests a similar *F* and analogous short-range magnetic correlations at *T* ≫ *T*_N_, which may account for the observed hysteresis of the ZFC-FC curves when recorded at low field.

**Figure 7:**
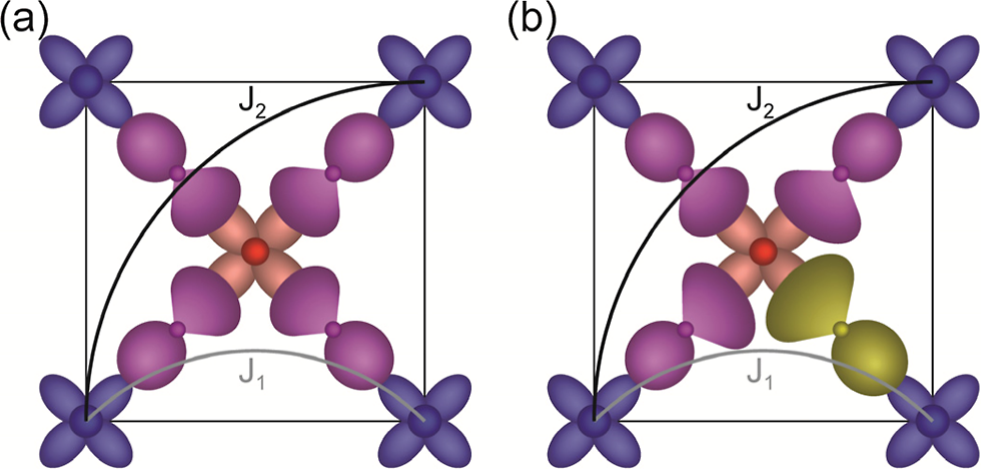
Sketch illustrating the *J*_1_, *J*_2_ magnetic interactions in basal plane of fcc double perovskite structure for (a) Ba_2_MnWO_6_, where *J*_1_ is ruled by 2p(O)–2p(O) overlapping, and (b) Ba_2_MnWO_4.42_N_1.58_, where the larger overlapping of 2p(O)–2p(N) contributing to *J*_1_ is illustrated. Yellowish and purple orbitals represent 2p_*x*_–N and 2p_*x*_–O orbitals, respectively. Blue and orange orbitals illustrate 3d (*x*^2^–*y*^2^) orbitals of Mn^3+/4+^ and W^6+^, respectively.

Remarkably, the frustration parameter in BMWON is found to be much larger: *f* ≈ 18.4. According to the previous discussion, it follows that *F* (≈*J*_1_/*J*_2_) is expected to be larger than ≈1.06 as determined in BMWO. In double perovskites with symmetry lower than *Fm*3̅*m*, large octahedral rotations or the coordination in the MX_6_ octahedra have been identified to be correlated with frustration (*f*). Here, the cubic nature of the investigated compounds excludes octahedral rotations and thus frustration should be of primary electronic origin rather than a consequence of geometric effects. Therefore, as *f* (BMWO) ≪ *f* (BMWON), we anticipate *F*(BMWON) > *F*(BMWO). To rationalize this prediction, we take into account early suggestions^29^ and recent first principle calculations performed by Katukuri et al.^2^ in the related double perovskites Sr_2_CuTeO_6_ and Sr_2_CuWO_6_. These authors showed that among the interactions contributing to *J*_1_, the direct Mn–O–O–Mn plays the most relevant role, due to the 2p(O)–2p(O) overlapping (Figure 7a). In the present oxynitride, the superexchange Mn–(O/N)–(O/N)–Mn is expected to be larger than Mn–O–O–Mn due to the larger matrix overlapping on Mn(3d)–N(2p)–N(2p)–Mn(3d) or Mn(3d)–N(2p)–O(2p)–Mn(3d) orbitals (Figure 7b), which are instrumental for a larger *J*_1_ and correspondingly a larger frustration in BMWON, as observed. Large *F*(BMWON) and *f*(BMWON) values anticipate a small fraction of ordered magnetic moments at the lowest temperature. Indeed, this is in agreement with our experimental results: from magnetic neutron diffraction data, we determined μ = 0.48 μ_B_ in BMWON.

## Conclusions

The topochemical ammonolysis at moderate temperatures of B-site ordered Ba_2_MnWO_6_ using high ammonia flow rates produces the new double perovskite oxynitride Ba_2_MnWO_4.42_N_1.58_ with high cation order, crystallizing in the same space group as the oxide, *Fm*3̅*m*, but with an expanded unit cell parameter. This process induces the oxidation of Mn^2+^ cations to Mn^3+^/Mn^4+^ which, together with the increase in the covalency of bonding caused by the substitution of oxygen by nitrogen, originates dramatic changes in the magnetic properties of the double perovskite. The magnetization data of Ba_2_MnWO_6_ show that its effective moment is larger than expected for only Mn^2+^. We have tentatively interpreted this observation, which is in agreement with earlier reports, on the basis of the covalency of the W(5d)–O(2p) bond and the associated charge transfer, which is further exacerbated in the oxynitride due to the smaller electronegativity of nitrogen compared to oxygen. The study of the magnetic and neutron diffraction data of Ba_2_MnWO_4.42_N_1.58_ shows that the nitridation leads to an enhanced frustration in the fcc lattice that we attribute to a covalency-induced reinforcement of the *J*_1_ antiferromagnetic interactions between nearest neighbor magnetic ions in the fcc structure. It has been recently reported that lattice distortions and orbital filling of the diamagnetic metallic ions in A_2_MnB″O_6_^2,4^ offer a route to tune the magnetic frustration. Here, we have shown that the O/N anionic substitution offers a genuine electronic alternative to change the frustration. This finding is reminiscent of recent reports on enhanced frustration in the magnetic nitride spinel MnTa_2_N_4_^6^ and suggests a path toward materials with tailored magnetic interactions.

## Supporting Information Available

X-ray diffraction pattern of the oxide precursor prepared by the citrate method; refinement of SXRPD data of Ba_2_MnWO_6_ and Ba_2_MnWO_4_N_2_; structure parameters and electron diffraction patterns of Ba_2_WMnO_4.64_N_1.36_; M(H) measurements of Ba_2_MnWO_6–*x*_N_*x*_ samples; discussion on the selection of the temperature range for susceptibility fitting; and discussion on role of covalency in magnetic properties (PDF)
