# Supplementary material for: Nitride Tuning of Magnetic Frustration in the Double Perovskite Ba2MnWO6
Source: Chem Mater. 2024 Oct 9;36(20):10267–75. doi: 10.1021/acs.chemmater.4c02114 (PMC11925330; doi:10.1021/acs.chemmater.4c02114)
Supplement: Supplementary file 1 — cm4c02114_si_001.pdf [file cm4c02114_si_001.pdf]

Supplementary Information

Nitride tuning of magnetic frustration in the double perovskite Ba_2MnWO_6

Judith Oró-Solé, Carlos Frontera, Jhonatan R. Guarín, Jaume Gàzquez, Bernat Mundet, Clemens Ritter, Josep Fontcuberta and Amparo Fuertes**

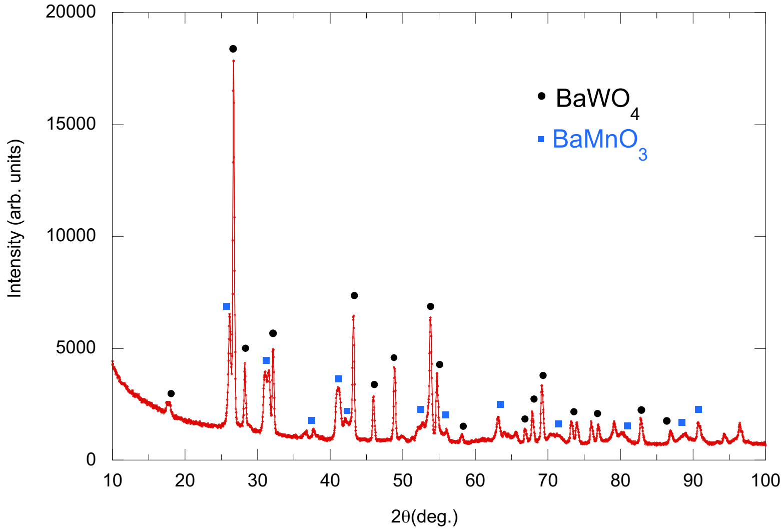

Figure S1. X-ray diffraction pattern (λ Cu Kα= 1.5418 Å) of the oxide precursor obtained by the citrate method showing the mixture of ternary oxides $BaMnO_3$ and $BaWO_4$.

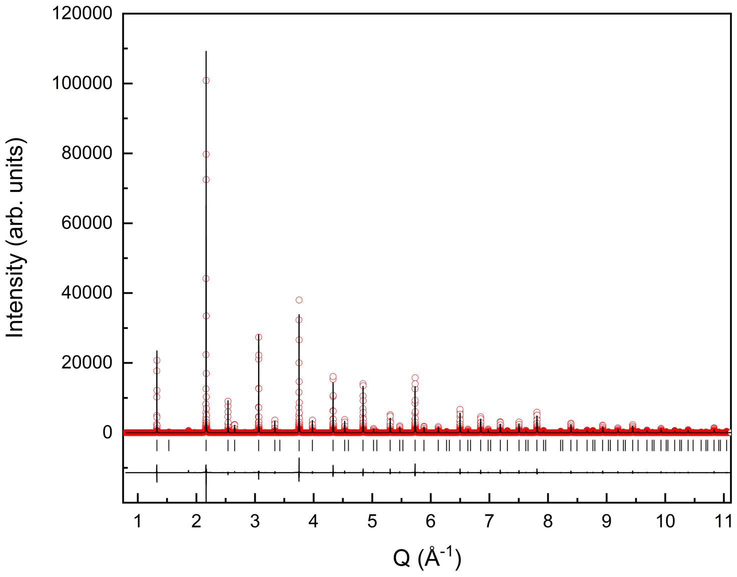

Figure S2. Rietveld fit to synchrotron X-ray powder diffraction pattern (λ=0.41385 Å) of Ba_2MnWO_6 performed in the space group *Fm-3m* with parameter a=8.20337(1) Å.

Table S1. Summary of the *Fm-3m* model refined against room temperature synchrotron X-ray powder diffraction data for Ba_2MnWO_6 (λ= 0.41385 Å). Refined cell parameter and agreement factors: a=8.20337(1) Å. R_{Bragg}= 4.0 %, R_{wp}= 11.4 %.

Atom	Site	*x*	*y*	*z*	B(Å^2)	Occupancy
Ba	*8c*	0.25	0.25	0.25	0.497(8)	1
Mn	*4b*	0.5	0.5	0.5	0.36(2)	1
W	*4a*	0	0	0	0.175(4)	1
O	*24d*	0.23441(3)	0	0	0.58(4)	1

Bond	d(Å)	Bond	d(Å)	Bond	d(Å)
Ba-O	2.903(2)x12	Mn-O	2.179(3)x6	W -O	1.923(3)(x6)

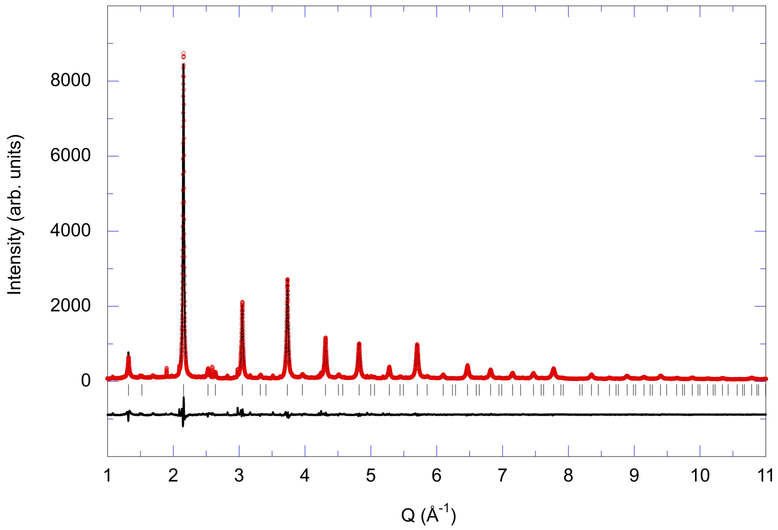

Figure S3. Rietveld fit to synchrotron X-ray powder diffraction pattern (λ=0.41385 Å) of $Ba_2MnWO_4N_2$ performed in the space group *Fm-3m* with parameter a=8.23911(5) Å.

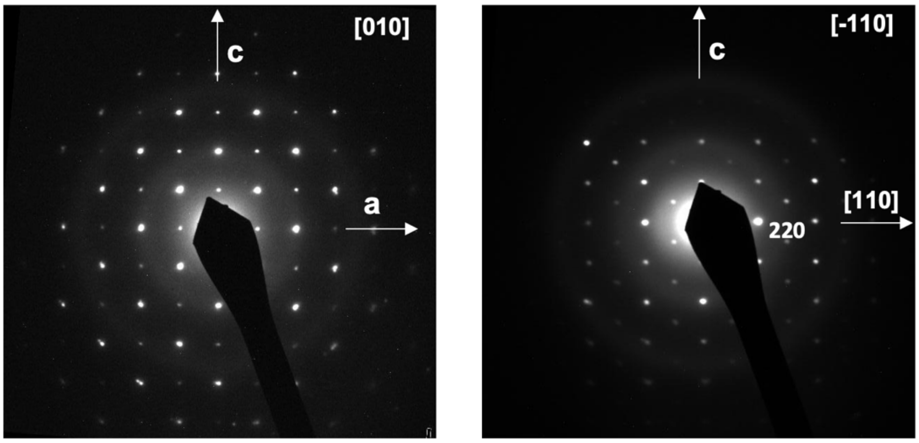

Figure S4. Selected electron diffraction patterns of $Ba_2WMnO_{4.64}N_{1.36}$.

Table S2. Summary of the *Fm-3m* model refined against room temperature synchrotron X-ray powder diffraction data for $Ba_2MnWO_{4.64}N_{1.36}$ (λ= 0.41385 Å). Refined cell parameter and agreement factors: a=8.24144(4) Å. R_{Bragg}= 3.35 %, R_{wp}= 11.6 %. [a]

Atom	Site	*x*	*y*	*z*	B(Å^2)	Occupancy
Ba	*8c*	0.25	0.25	0.25	0.78(3)	1
Mn1/W1	*4b*	0.5	0.5	0.5	1.05(2)	0.974(4)/0.026
W2/Mn2	*4a*	0	0	0	1.10(8)	0.974/0.026
O/N	*24d*	0.2370(6)	0	0	1.30(9)	0.773/0.227

Bond	d(Å)	Bond	d(Å)	Bond	d(Å)
Ba-O/N	2.916(3)x12	Mn1/W1-O/N	2.167(5)x6	W2/Mn2-O/N	1.953(5)x6

[a] The O/N occupancies were constrained to the anion composition obtained by chemical analysis.

Section S1. On the selection of the temperature range for magnetic susceptibility temperature dependence fitting

This is a subtle issue that may lead to spurious results, as discussed below.

We first stress that the fits have been done down to around 25 K, significantly above the temperature where long range magnetic order is stablished (< 8.5 K in all cases).

On the other hand, data for all samples show some departure from Curie Weiss behavior (this is why we describe it in the main text as displaying "a roughly Curie-Weiss behavior"), even when a temperature independent susceptibility term is included in the fits. In the following we present the results from using different fitting temperature ranges and we elaborate on them.
We have performed additional fits limiting the lowest temperature to 150 K, thus fitting the 150-300 K range. It turns out that the extracted moments from the high temperature fits (see Table below) are significantly different from those obtained using a larger temperature (≈ 30-300 K):

Fitting temperature range	$Ba_2MnWO_{4.42}N_{1.58}$	Ba_2MnWO_6
30-300 K (reported in the paper)	5.16 μ_B	6.28 μ_B
150-300 K	6.4 μ_B	7.1 μ_B

As expected, data in this Table illustrate the relevance of choosing the appropriate temperature range for data fitting. Inspection of these data indicates some relevant points:

- We first note that in all cases the values obtained from the fits in the high temperature range follow the same trend that those obtained from the low temperature range and both are larger than those expected from the single Mn^{m+}

ions, as described in the paper. Therefore, the observation and discussion that we included in the main text regarding the role of covalency, remains valid.

- Still the observation of a larger moment when reducing the fitting range to the high temperature part of the susceptibility curve may be puzzling. An insight into the physical origin of this observation can be obtained from inspection of the evolution of the temperature-independent χ_0 term in $\chi(T) = \frac{C}{T-\theta_{CW}} + \chi_0$, extracted from measurements recorded at different magnetic fields and using different temperature ranges for the fits. In the Table below we include the extracted χ_0 term (emu/mol) for both samples:

Compound	50 Oe	500 Oe	5 kOe (30-300K)	5 kOe (150-300K)
Ba_2MnWO_6	NA	2.3x 10^{-4}	-2.4 x10^{-3}	-4.6 x10^{-3}
$Ba_2MnWO_{4.42}N_{1.58}$	1.26 x10^{-3}	3.6 x10^{-4}	-1.9 x10^{-3}	-4.0 x10^{-3}

Data in this Table clearly evidences that when increasing the magnetic field, the diamagnetic contribution to χ_0 emerges, but it is masked by a ferromagnetic-like ($\chi_0 >$ 0) contribution that dominates at lower fields. The diamagnetic-like component is more obvious when increasing the magnetic field as its magnetization increases (in absolute value) linearly with magnetic field being a ferromagnetic contribution (M_{FM}) likely saturated ($\chi_0 = \chi_{Diam.} + {}^{M_{FM}}/_{H}$).

These data, together with the magnetization loops M(H), show that the measured susceptibility actually contains magnetization responses coming from: a) a paramagnetic component with a hyperbolic ($1/(T-\theta_{CW})$) temperature dependence, b) a diamagnetic component (basically T-independent), and c) a tinny ferromagnetic component that saturates at large magnetic field and shall decrease with temperature. Therefore, to emphasize the paramagnetic ($\approx 1/T$) contribution to the measured $\chi(T)$, the fits expanding down to the lowest possible temperature (obviously being at >> T_N) are expected to produce the best estimate of the paramagnetic moments in the system. This is why, in our view, data collected in the 30-300 K range better represents the magnetic moments of Ba_2MnWO_6 and $Ba_2MnWO_{4.42}N_{1.58}$. These are the data included in the paper.

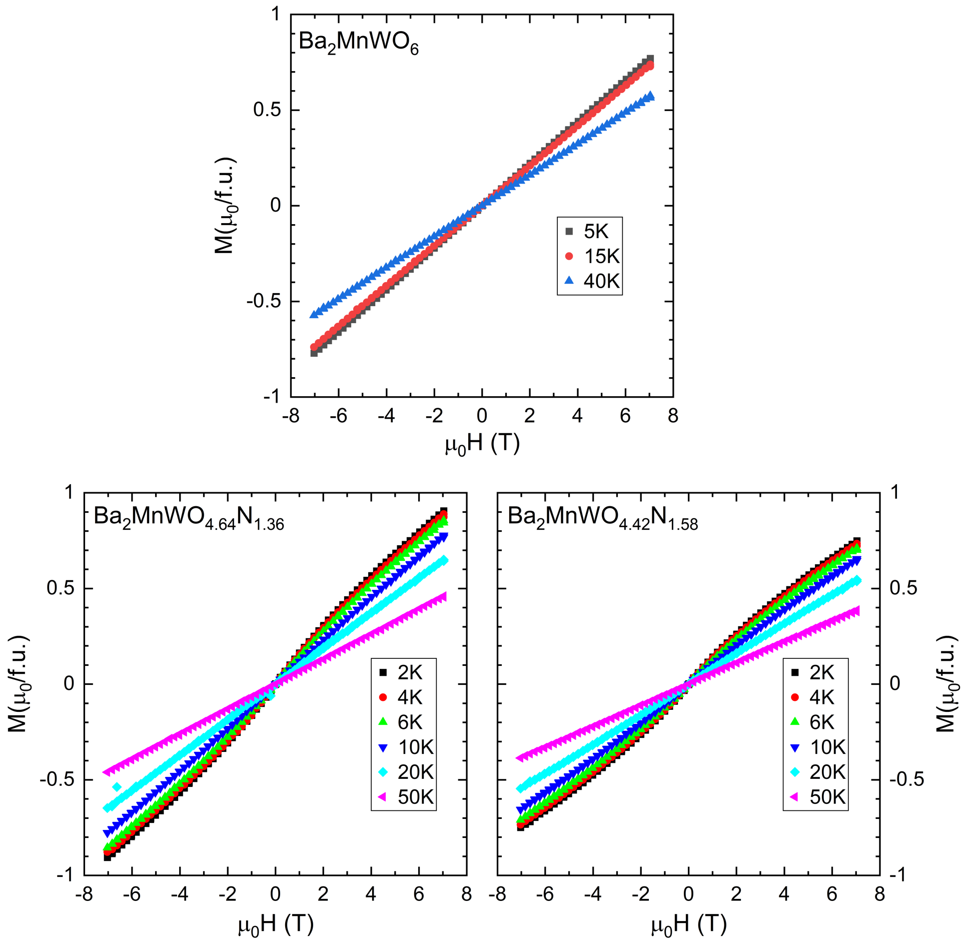

Figure S5. Magnetization M(H) curves at various temperatures for: Ba_2MnWO_6(top), $Ba_2MnWO_{4.64}N_{1.36}$(bottom left) and $Ba_2MnWO_{4.42}N_{1.58}$(bottom right).

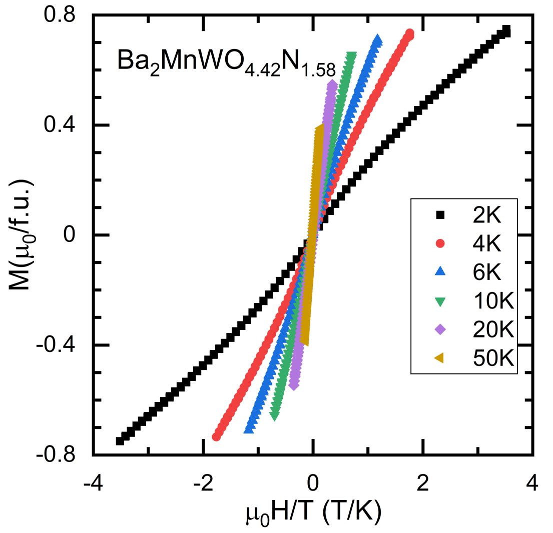

Figure S6. Magnetization of $Ba_2MnWO_{4.42}N_{1.58}$ at various temperatures and fields plotted vs H/T.

Section S2. On the role of covalency on magnetic properties of related compounds (the WO_3 case).

The role of covalency in magnetic moments in simple oxides such as MnO is well documented, as mentioned in the manuscript. As the covalency of bonds is larger for nitrogen than for oxygen, a larger contribution in the oxynitride is to be expected.

In the case of W, having more extended orbitals than Mn, the effect should be also present in W-O compounds. In the particular case of WO_3, for instance, we note that stoichiometric WO_3 is a semiconductor and its structure is strongly dependent on temperature. Taking the most common monoclinic phase for the present discussion, recent calculations have shown that W-O covalency is as large as 11 % [1], which is consistent with earlier conclusions derived for a cubic structure of WO_3 [2]. Typically, WO_3 is an n-doped semiconductor due to the presence of oxygen vacancies. It thus follows that the associated magnetic moment of W^{5+} should be present (and indeed observed, by E. K H Salje et al [3]. Disentangling covalency effects from the extrinsic n-doping on the formation of a magnetic moment on W^{m+} is probably beyond experimental capabilities.

[1] Trioni, M.I; Cargnoni, F.; Americo, S.; Soave, R. New Insight into the Electronic and Magnetic Properties of Sub-Stoichiometric WO_3: A Theoretical Perspective. *Crystals* **2024**, 14, 372.
[2] Hjelm, A.; Granqvist, C.G.; Wills, J.M. Electronic Structure and Optical Properties of WO_3, $LiWO_3$, $NaWO_3$, and HWO_3. Phys. Rev. B 1996, 54, 2436[3] Salje, E.K.H; Rehmann, S.; Pobell, F.; Morris, D.; Knight, K.S.; Herrmanndörfer, T.; Dove, M.T. Crystal Structure and Paramagnetic Behaviour of ε-WO_{3-x}. *J. Phys.: Condens. Matter.* **1997**, 9, 6563–6577.
